# Global Trade Comes Home: Community Impacts of Goods Movement

**DOI:** 10.1289/ehp.116-a78

**Published:** 2008-02

**Authors:** Andrea Hricko

For many U.S. residents, 2007 was a year of heightened awareness of some of the problems of global trade. Extensive recalls of melamine-tainted pet food in the spring followed by even larger toy recalls in the summer and fall raised consumer concerns about how the United States can ensure the safety of products shipped in from overseas. The *Salt Lake Tribune* and the *Wall Street Journal* detailed injuries and illnesses threatening the health of Chinese workers making products for export to the United States. And on 15 December 2007, a *New York Times* feature detailed the practice of farming fish in toxic Chinese waters for export to the United States and other countries.

While these news stories demonstrate some of the pitfalls of globalization, much less attention has focused on air pollution and other community-level impacts in the United States, as toys, electronics, food, and other imports travel through ports, then to trucks, trains, warehouses, and stores in a complex system called “goods movement.” Along the route, residents are exposed to diesel exhaust and other vehicle emissions, noise from truck-congested roads, bright lights from round-the-clock operations, and other potential health threats.

Transportation experts refer to these impacts simply as “externalities” of transport, but to community residents they can directly harm the quality of daily life. As ports and goods movement activity expands throughout the United States, a major challenge is how to make its health and community impacts a more central part of policy discussions.

## Economic Benefits, Community Costs

Economic development advocates call the side-by-side ports of Los Angeles and Long Beach Southern California’s “economic engine.” Combined, they handle the most containers of any U.S. port. With more than 40% of all imports for the entire United States coming through the Los Angeles/Long Beach port complex, according to the U.S. Department of Transportation, the ports are critical to the national economy. A March 2007 national economic impact study by the twin ports reported that imports coming through the complex generated jobs, income, and tax revenue in every state of the nation.

While recognizing the economic importance of international trade, the U.S. Environmental Protection Agency (EPA) has called the movement of freight a “public health concern at the national, regional and community level.” In a 22 August 2007 *Federal Register* announcement of a meeting of its National Environmental Justice Advisory Council (NEJAC), the EPA also described mounting evidence that local communities adjacent to ports and heavily trafficked goods movement corridors are the most significantly impacted by the goods movement system.

The ports of Los Angeles/Long Beach combined contribute more than 20% of Southern California’s diesel particulate pollution and are the single largest source of pollution in Southern California, according to the South Coast Air Quality Management District (AQMD), the region’s air quality regulatory agency. The California Air Resources Board (CARB), in its 2006 *Emission Reduction Plan for Ports and Goods Movement*, calculated that in California alone there are 2,400 premature heart-related deaths related to port and goods movement pollution, 62,000 cases of asthma symptoms, and more than 1 million respiratory-related school absences every year. Nationwide, reports James Corbett of the University of Delaware and colleagues in the 15 December 2007 issue of *Environmental Science & Technology*, an estimated 60,000 lives are lost prematurely every year due to ship emissions, which are virtually unregulated.

Recent research findings about living close to traffic emissions add to concerns. A study by investigators at the University of Southern California (USC), published 17 February 2007 in *The Lancet*, showed that children living near freeway traffic had substantial deficits in lung function development between the ages of 10 and 18 years, compared with children living farther away. “Since lung development is nearly complete by age eighteen,” says lead author W. James Gauderman, “an individual with a deficit at this time will probably continue to have less than healthy lung function for the remainder of his or her life.”

Other studies published in the February 2003 and September 2005 issues of *EHP* linked traffic exposure to increased risk for low birth weight and premature birth. A new study published 6 December 2007 in the *New England Journal of Medicine* showed that adults with asthma who spent just 2 hours walking on a street with heavy diesel traffic suffered acute transient effects on their lung function along with an increase in biomarkers that indicate lung and airway inflammation. In addition, research by the EPA-funded Southern California Particle Center at the University of California, Los Angeles, published in the April 2003 issue of *EHP*, demonstrated that ultrafine particles from incomplete combustion of engine fuels and lubricating oils can bypass the body’s defense mechanisms, gain entry to cells and tissues, and alter or disrupt normal cellular function.

## Regulation to Date

In 2005, CARB issued guidelines that recommend avoiding construction of new schools and homes within a mile of a railyard or 500 feet of a busy highway. A few years earlier, California legislators, citing health effects research findings, passed SB 352, a law prohibiting building new schools within 500 feet of a busy road or freeway. But the 2003 law permits several loopholes, such as allowing a school district to show that it is able to mitigate traffic emissions so that pupils and staff will suffer no significant health risk. The law also requires that a school district verify that any railyard within a quarter mile of a new school will not present a public health threat. Some school districts, in the scramble to build new facilities, are continuing to site new schools near freeways and rail operations.

Conversely, railyards and freeways also continue to be proposed in close proximity to schools and homes, such as a proposed truck expressway to speed trucks away from the Southern California ports, which would pass within 100 feet of homes and 700 feet of a local school. The draft environmental impact statement (EIS) for the project, issued in August 2007 by the California Department of Transportation (Caltrans) acknowledges the scientific research: “Some recent studies have reported that proximity to roadways is related to adverse health outcomes—particularly respiratory problems.” But the EIS goes on to say that using these studies to determine if there will be adverse impacts from the truck expressway project is premature.

According to Ron Kosinski, deputy district director for the Caltrans district covering Los Angeles County, the Federal Highway Administration (FHWA) is delaying any policy decisions related to health effects from proximity to traffic until the conclusion of a review of all the studies by the Health Effects Institute—a report that is not expected for several years. FHWA spokesman Doug Hecox says, “[The agency is] not suggesting that nothing should be done. But there are no conclusive studies right now drawing a direct relationship between the number of trucks on a road and the percent of impairment of an affected child.”

Environmental, community, and public health groups have long pressured Los Angeles and Long Beach port authorities to take action on port pollution. In 2006, an historic agreement called the Clean Air Action Plan (CAAP) was signed, vowing that the ports would reduce air pollution by 45% within the next 5 years. However, some community and environmental groups are concerned that the deadlines set in the CAAP are slipping.

Port of Los Angeles executive director Geraldine Knatz responds that the CAAP “is a five-year process that requires major investment in construction and new equipment, and in the interim, cargo movement through our ports continues.” Knatz also points to a new program to reduce port-related truck emissions by 80% by 2012—a $2 billion initiative that she says “cannot simply happen overnight.” In December 2007, both ports adopted container fees to fund the replacement of 17,000 polluting big-rig trucks with new models that meet tighter EPA diesel emission standards.

At the state level, CARB issued new rules in December 2007 that would require ships to plug in to electricity rather than using diesel auxiliary engines when docked in the harbor and that would require stricter emissions standards for trucks frequenting ports and railyards. The South Coast AQMD has long championed stricter controls on ports and rail operations to protect public health, as well as environmental justice considerations. In 2006 the agency issued rules to reduce pollution from idling locomotives in railyards, but railroad companies sued to block them. In 2007 a Los Angeles–based U.S. District Court judge struck down the agency’s rules, arguing that it lacked authority to adopt them; the agency is appealing the decision.

According to the South Coast AQMD, emissions from ships are also underregulated, with no significant international or federal emission control regulations. In 2004, the EPA announced plans to put in place new standards for ships and locomotives. On 15 January 2008, the Greenwire news service reported these standards were under review at the White House Office of Management and Budget, which must approve them before the EPA can sign off on them.

## Increased Trade Expected

The health and environmental justice impacts of port, rail, and trucking pollution are not limited to California. In South Carolina, for example, environmental groups and homeowners are troubled by anticipated impacts of a proposed terminal expansion at the old Charleston Navy Base, which the South Carolina Coastal Conservation League says will triple the container volume through Charleston and generate thousands more truck trips a day through a low-income black neighborhood. “An access road and off-ramp will go right through our Rosemont community as trucks leave the port terminal for the nearby interstate highway,” New Rosemont Neighborhood Association president Nancy Button told participants of a recent community–academic conference on port health impacts held in Los Angeles.

According to *The Journal of Commerce Online* (*JoC*), a news magazine covering international trade and goods movement, many U.S. ports are expanding in hopes of capitalizing on rising international trade volumes. Historically, says maritime industry economist Bill Ralph, as quoted in the 16 January 2008 *JoC*, international container trade in the United States has an annual growth of about 7%. In 2006, U.S. containerized imports grew by 11%. But in 2007, says Ralph, they increased by only 3%, due to a slowdown in the housing and auto markets. Economist Walter Kemmsies, quoted 2 days earlier in the *JoC*, predicts that U.S. container trade will return to its normal 7% annual growth within the next 2 years and continue to grow steadily—even faster if the United States enters into more free trade agreements.

The EPA Office of Environmental Justice (OEJ) has taken note of the growth trends and the rising environmental health concerns about port and goods movement expansion. In August 2007, acting OEJ director Charles Lee appointed a new working group to study the impacts of ports and goods movement through an “environmental justice lens,” with a report expected in June 2008. Land use decision making will be 1 element in the report, along with community participation, regulatory mechanisms, innovative technologies, and more.

Projected increases in foreign trade, along with many states’ planned expansion of highways, rail facilities, and ports to handle Asian imports, cause concern about increased air pollution if regulations to reduce emissions do not keep pace with trade growth. In the 22 August 2007 *Federal Register*, the EPA noted that the anticipated increase in trade will have air quality impacts, and the agency threw out a challenge to the ports and companies involved in goods movement: “It is becoming increasingly important that these entities operate sustainably, i.e., economically viable, environmentally and socially responsible, safe and secure.”

## Community Response

As this global goods movement system expands, communities across North America are now recognizing that they are facing similar circumstances and common conflicts. And they are banding together, in small and large coalitions, to address the impacts.

In the 1990s, just a few groups such as the Sierra Club, the Environmental Health Coalition, the OEJ, and homeowners near the ports were focused on the effects of the global supply chain. But 2001 turned out to be a watershed year. That year, the Natural Resources Defense Council, the Coalition for Clean Air, Communities for a Better Environment, and 2 harbor-based homeowner’s associations filed a lawsuit challenging the Port of Los Angeles’s environmental review of planned construction for a major shipping terminal. Two years later they won a $50 million landmark settlement from the city requiring environmental mitigations, such as the “plug in” rule issued by CARB in December. A new era had begun—one that started to shift public attention from the role of international trade simply as the region’s major economic engine to the potential perils of uncontrolled goods movement expansion.

That same year, the NIEHS-funded Southern California Environmental Health Sciences Center, based at USC, held a town meeting to share its research findings with community groups, residents, workers, and policy makers. In turn, scientists heard the emerging concerns of residents about diesel emissions near the ports, railyards, and warehouses. Research findings on the health impacts of air pollution soon began to find their way into policy debates on goods movement and port expansion.

Over the next 5 years, multiple partnerships started to come together to specifically address issues of ports and goods movement in California. Among the collaborative efforts active today are the Ditching Dirty Diesel Collaborative based in Oakland, aimed at developing a regional strategy to reduce diesel emissions; the Trade, Health & Environment (THE) Impact Project, a community–academic collaborative aimed at elevating community voices in the goods movement policy debate and using science-based information to inform public policy; the Port Work Group of Green LA, which aims to ensure that the Port of Los Angeles becomes truly green, with the support of the city’s mayor; and a broad-based coalition aimed at improving wages and working conditions (including less-polluting vehicles) for port truck drivers.

Elsewhere, residents in a neighborhood near the Port of Seattle have been counting big-rig trucks parked overnight in their community in an effort to keep port-related pollution, safety hazards, and blight out of their neighborhoods. In Arizona, a school superintendent has asked officials not to enact zoning changes that would allow construction of a major intermodal facility (a railyard at which cargo is transferred between trucks and trains) across the street from a local elementary school. And on Long Island, residents are asking the state of New York to reconsider its plans to build an intermodal facility near residential communities and a wildlife preserve.

## Tools for Action

Many groups impacted by ports and goods movement came together in late 2007 at Moving Forward, the first North American community-oriented gathering on this topic, which was organized by THE Impact Project and cosponsored by private groups along with NIEHS- and EPA-funded centers.

Participants shared information on current health research related to goods movement, community concerns about health impacts, future goods movement expansion projects (such as plans to deepen the harbor at the Port of Savannah, Georgia, to handle larger ships carrying twice as many containers), and community efforts to effect change. Presenters described tools for action, such as methods for mapping goods movement activities in communities; understanding who the key goods movement stakeholders and decision makers are; ways to incorporate credible, current scientific research findings into educational and policy efforts; and new methods for developing health impact assessments.

Eric Kirkendall from Kansas was struck by the commonalities at the conference. Back home, he had formed the Johnson County Intermodal Coalition in response to proposals to build an intermodal railyard near the small town of Gardner and surround his 4-acre homestead on 3 sides with 12-acre warehouses. Kirkendall says, “We sometimes feel alone in Kansas. But by the end of the conference I understood that we are not alone. We have much to share with, and learn from, other groups with similar challenges, as well as from scientists and policy makers.”

Some attendees thought more attention should be focused on American consumer habits, a point echoed by Rev. Peter Laarman, executive director of Progressive Christians Uniting. He urges a closer look at the hidden costs of imports. “Americans think of themselves as consumers rather than as citizens,” he says. “We don’t care, for example, if Chinese workers toil in factories with no safety regulations, or if residents in communities near our ports have to breathe dirtier air. What we care about is ‘How much do I have to pay for an iPod?’ and ‘Where can I buy this doll for under ten dollars?’”

By their very nature, the ports and goods movement debates faced by community groups throughout North America can help to inform future discussions about consumerism and globalization. As far as health effects go, however, research findings and community experience are strongly suggesting that global trade, while an apparent boon to our economy, will continue to pose a serious threat to our population’s environmental health unless protective and collective action is taken, and soon.

## Figures and Tables

**Figure:**
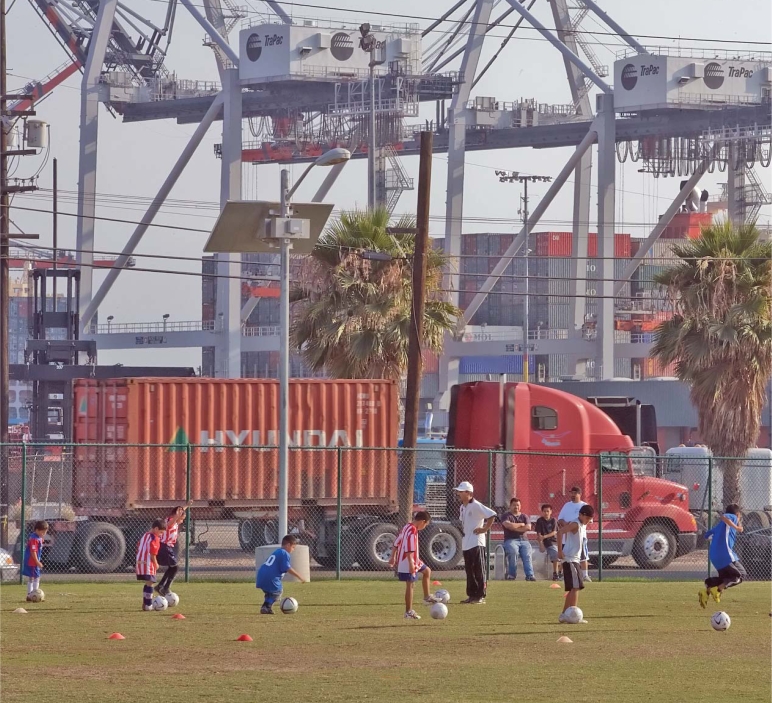
Children play soccer next to the TraPac terminal at the Port of Los Angeles, Wilmington, California.

