# Errata

**Published:** 2008-03

In the February Spheres of Influence article [Environ Health Perspect 116:A78–A81 (2008)], the first sentence of the second paragraph under the subhead “Community Response” should have read “In the 1990s, just a few groups such as the Sierra Club, the Environmental Health Coalition, the Center for Community Action and Environmental Justice, and homeowners near the ports were focused on the effects of the global supply chain.”

In the February Science Selection article “Exposure Under Pressure: Lead Linked to Release of Cortisol in Children” [Environ Health Perspect 116:A83 (2008)], the range of the known prenatal blood lead levels was ≤ 1.0–6.3 μg/dL.

*EHP* regrets the errors.

